# PHYSICAL ACTIVITY, HEART RATE VARIABILITY, FRAILTY, AND COMPLICATIONS IN GYNECOLOGIC ONCOLOGY

**DOI:** 10.1093/geroni/igae098.0129

**Published:** 2024-12-31

**Authors:** Emma Barber, Olivia Foley, Connor Wang, Allison Grubbs, Karl Bilimoria

**Affiliations:** Northwestern University, Chicago, Illinois, United States; Northwestern University, Evanston, Illinois, United States; Northwestern University, Evanston, Illinois, United States; Rush University, Chicago, Illinois, United States; University of Indiana, Indianapolis, Indiana, United States

## Abstract

Gynecologic oncology patients undergo complex operations with high rates of postoperative complication. Frailty is consistently associated with complication but is not routinely measured in clinical practice. This study aimed to examine the association of physiologic and activity data with frailty and postoperative complications in presurgical gynecologic oncology patients. We performed a prospective cohort study of gynecologic oncology surgery patients who wore wearable actigraphy rings before and after surgery from 03/2021-11/2023. Exposures were physiologic data (heart and respiratory rates, heart rate variability), preoperative activity intensity and level. Primary outcomes included frailty (Pajewski frailty score) and 30-day complications. Multivariable models were adjusted for age, cancer and surgery type. We enrolled 96 patients and 87 met inclusion criteria. 41.3% were not frail, 44% were pre-frail and 14.7% were frail. Average heart rate and respiratory rate differed between frail and non-frail patients (HR 78.6 vs 68.3bpm, aOR=1.10,95%CI1.01-1.20; RR 18.6 vs 16.7b/m,aOR=2.1,95%CI1.20–3.55). Heart rate variability (RMSSD) also differed (24 vs 30.9ms,aOR=0.81,95%CI0.68-0.97). Preoperative physical activity was also associated with postoperative complications. Overall, 29.9% experienced a complication and higher activity was associated with fewer complications: 500-1000 MET-mins (OR=0.26,95%CI=0.07-0.92) and >1000 MET-mins (OR=0.25,95%CI=0.07-0.94) vs <500 MET-mins; ≥8000 steps (OR=0.25,95%CI=0.08-0.73) vs <8000 steps. In conclusion, physiologic data and activity levels differ in frail compared to not frail patients independent of age. Wearable devices may be useful in the preoperative assessment of frailty. Furthermore, higher preoperative activity levels are associated with fewer postoperative complications. Physical activity may be a modifiable risk factor for adverse postoperative outcomes.

